# Research on the Formation Mechanism of Health Insurance Fraud in China: From the Perspective of the Tripartite Evolutionary Game

**DOI:** 10.3389/fpubh.2022.930120

**Published:** 2022-06-23

**Authors:** Yun Fei, Yi Fu, Dong-xiao Yang, Chang-hao Hu

**Affiliations:** ^1^The Second Xiangya Hospital of Central South University, Changsha, China; ^2^School of Business, Central South University, Changsha, China; ^3^College of Business Administration, Krirk University, Bangkok, Thailand

**Keywords:** health insurance fraud, defraud reimbursement, tripartite evolutionary game, regulatory measures, formation mechanism

## Abstract

The problem of fraud in China's health insurance has existed for a long time and is becoming more serious, which needs to be solved urgently. This article constructs a tripartite evolutionary game model to study the formation mechanism of the Health Insurance fraud, game participants including medical administrative organization (MAO, a government department responsible for health insurance supervision), medical institutions (MI, such as hospitals), and insured individuals (II, who participating in medical and healthcare insurance). By analyzing the equilibrium of the tripartite evolutionary game, this paper makes an in-depth study on the formation and resolution of health insurance fraud. The results show that: (1) How to prevent the fraud behavior of the medical institutions is the difficulty and core of the problem. It is necessary to achieve effective supervision of the MAO, improve the internal management of the MI and give play to the supervisory role of the II. (2) The regulatory behavior of the MAO needs to focus on protecting the interests of the II, not only to encourage them to actively play the role of supervision and reporting but also to prevent their collusion with MI. (3) On the one hand, the MAO needs to strengthen supervision and increase the punishment for fraud. On the other hand, they also need to take incentive measures to guide all subjects to form a sound internal management mechanism.

## Introduction

Health insurance fraud is a major challenge facing the insurance industry both in the developing and developed world, it has caused substantial and increasing costs in medical insurance programs (1). In recent years, China's medical and health insurance has been popularized and developed rapidly, and a relatively perfect medical insurance supervision and legal system have been formed. In 2021, the promulgation of the *Regulations on the administration of Health insurance* and *Medical Security Law (Draft for comments)* also provides a strong guarantee for the sustainable development of the health insurance industry. However, some problems in the field of medical and health insurance have become increasingly prominent, especially the problem of fraud is still pretty urgent. Making up false medical projects, over inspection and over treatment by hospitals and other illegal acts led to a large loss of China's health insurance. In order to vigorously crack down on such illegal acts, since September 2018, China's National Medical Insurance Bureau, together with the National Health Commission, has jointly launched a special action to crack down on insurance fraud, and dealt with a number of illegal cases in accordance with the law and regulations (2). The problem of fraud in health insurance has attracted great attention from all walks of life, and it is urgent to solve the problem in the field of health insurance.

Due to the supervision of health insurance is of great significance, in recent years, China's medical administrative organization has issued a number of documents to improve the health insurance legal system and solve the long-standing problem of fraudulent reimbursement. *The regulations on the administration of Health insurance*, which has been officially implemented since May 2021, clearly points out that it is necessary to strengthen the responsibilities of relevant subjects in the use of the health insurance fund and standardize the use of fund (3). On 16 June 2021, China's National Medical Insurance Bureau issued the *Medical Security Law (draft for comments)*, which defines the punishment measures for several violations of relevant laws such as infringement of health insurance (4). The improvement and promulgation of *Medical Security Law* will provide a legal basis for the promotion of national medical reform and restrict the behavior of subjects involved in health insurance fund, which is also of great significance to eliminate potential risks and ensure the safe use of funds. Hence, it can be seen that clarifying the relationship between the medical administrative organization, medical institutions, and insured individuals is very important to solve the problem of health insurance fraud.

The rest of this article proceeds as follows: The following section discusses the literature review, introducing the relevant research on the health and medical insurance fraud. The “Assumptions” section consists of the hypothesis of the game model and interpretations of notations. The “Tripartite Evolutionary Game Model of Health Insurance Fraud” section constructs an evolutionary game model between the MAO, MI and II. The “Simulation Analysis” section focuses on the influence of some important parameters on ESSs and reveals under what conditions the system will reach ESS. The “Conclusion” section concludes. Some meaningful suggestions are put forward in the “Suggestions for Government” section.

## Literature Review

In view of the problems of the health insurance fraud and fraudulent reimbursement, a large number of researchers conduct research by analyzing the problems, summarizing experience, studying the relationship and behavior of various subjects, evaluating the performance of health insurance fund operation, etc., and put forward solutions.

A lot of countries in the world have serious problem related to health insurance fraud, which has caused huge losses. For instance, in America, empirical evidence from New Jersey supports theories of hospital altruism (5), and healthcare fraud is a serious financial drain on the health-care system (6). Large numbers of cases have been investigated and prosecuted, resulting in the recovery of large dollar amounts. The Federal Bureau of Investigation (FBI) estimates that fraudulent billings to public and private healthcare programs are 3–10 percent of total health spending, or $75–250 billion in the fiscal year 2009 (7). And some researchers point that ~$700 billion is lost due to fraud, waste, and abuse in the US healthcare system (8). Another study finds that the over $2 trillion US healthcare system is ravaged by fraud, waste, and abuse, with an estimated one-third of all these costs frivolously spent in such ways (9). Despite increased funding and prosecution efforts by the government, healthcare fraud continues to be a major threat to the U.S. economy and public. While healthcare fraud will never be eradicated, specific efforts can be deployed to help rein in these complex fraud schemes (10).

In order to analyze the essence of health insurance fraud, Pande and Maas (11) provides a definitional understanding of healthcare fraud, defines specific frauds, categorizes healthcare frauds into four types and discusses efforts to mitigate healthcare fraud. Fraud can be seen in all insurance types including health insurance. And fraud in health insurance is done by intentional deception or misrepresentation for gaining some shabby benefit in the form of health expenditures (12). Furthermore, how to apply unsupervised outlier techniques at post-payment stage to detect fraudulent patterns of received insurance claims is an important issue (13). However, most available studies have focused on algorithmic data mining without an emphasis on or application to fraud detection efforts in the context of health service provision or health insurance policy (14). For instance, Yang and Hwang (15) proposes a data-mining framework that utilizes the concept of clinical pathways to facilitate automatic and systematic construction of an adaptable and extensible detection model. Thaifur et al. (16) uses the gaming theory to analyze the conditions and processes of the gaming between the hospital insured and the Urban Workers' Basic Medical Insurance organization and revealed the causes and existent necessity of the moral hazard. Sparrow and Malcolm (17) investigates whether there are any differences in public attitudes toward fraud committed against the public agencies vs. the private insurance companies. And the hazy boundaries of healthcare fraud has also been analyzed (18). King (19) has studied and summarized the practical experience of American Medical Insurance anti-fraud, and pointed out that the government should speed up the legislation of medical insurance anti-fraud, strengthen the fight against fraud, establish an anti-fraud data system and improve the level of anti-fraud technology. In addition, Zhou and Su (20) identifies and evaluates the risks of each stage of the medical insurance fund from the perspective of risk of breaches, summarizes the path of the medical insurance fund audit supervision so that that audit resources can be concentrated in important stages and key areas.

Now, few studies have focused on China's health insurance fraud that is an urgent problem to be solved. In this article, we not only deeply analyze the mechanism of health insurance fraud in China, but also put forward relevant suggestions. In fact, in the process of using and supervising the health insurance, there are mainly three types of participants: MAO, MI, and II. Each subject has different interest demands. By analyzing the interest of multiple subjects and the game relationship between them, we can get the causes and solutions of health insurance fraud. The innovation of this article is to comprehensively reveal the game relationship between the three subjects by constructing a tripartite evolutionary game model. Through the replication dynamic equation, this article investigates the strategy evolution trend of each subject, analyzes the main factors affecting the stability strategy, so as to explain the causes of health insurance fraud and put forward targeted solutions.

## Assumptions

**Figure 1:**
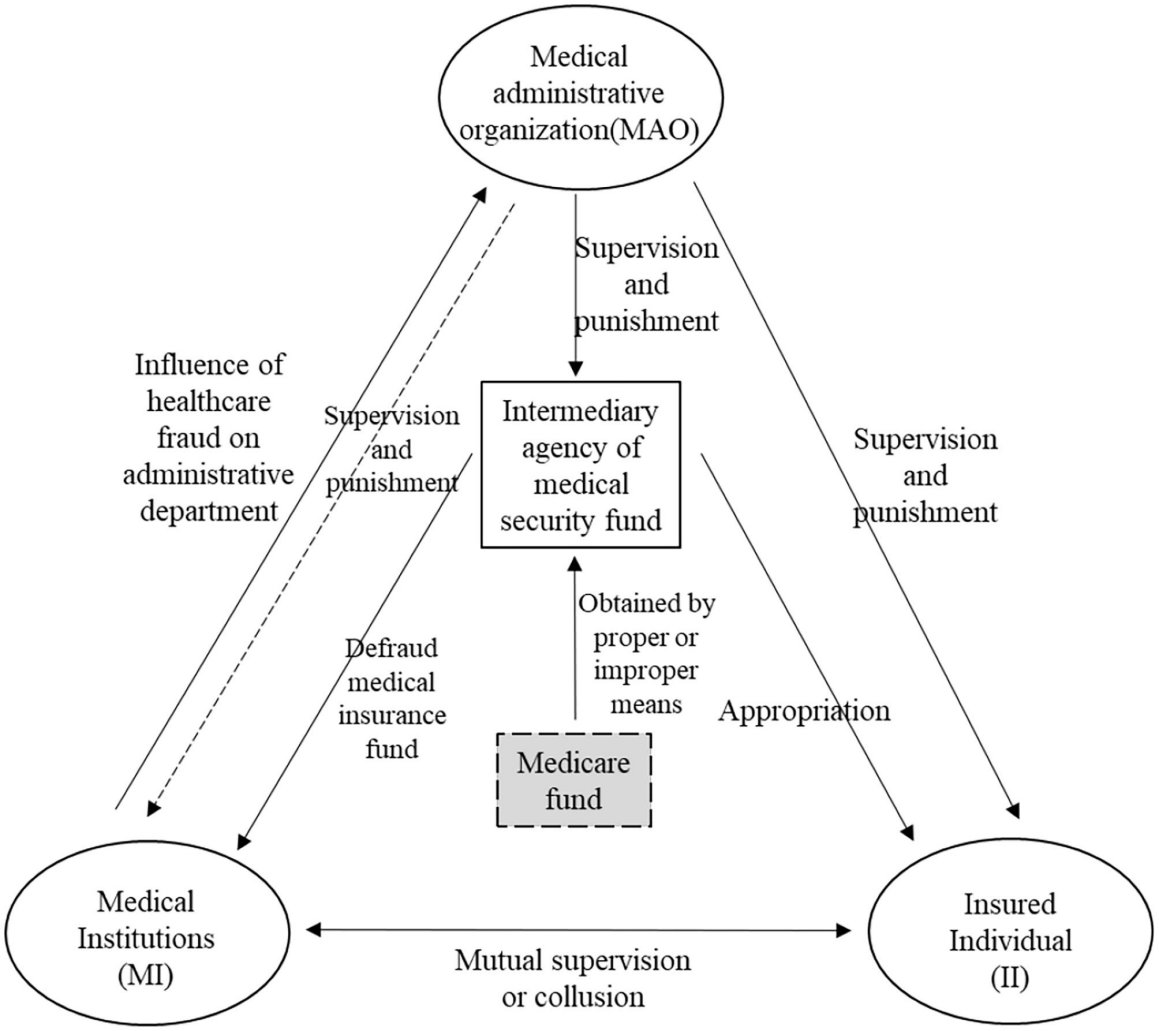
The relationship between the three subjects.

In order to build an evolutionary game model among the three subjects involved in the health insurance, it is necessary to clarify the relationship between the three subjects (see in Figure 1). Based on the practice of China's health insurance supervision, assumptions of game model are put forward as follows.

(1) The insured individuals only through collude with the medical institution to defraud the health insurance, but the latter does not necessarily collude with the individuals to fraud. Medical institutions can forge reimbursement vouchers and use individual accounts in violation of regulations. They have more professional ability, so they are easier to take fraud.

(2) The MAO shall give a certain reward (determined by ω) according to the self-correction strength of the medical institutions, and encourage them to improve their internal management system.

(3) The fraudulent behavior of the medical institutions has a huge negative impact on the MAO, and generally, the MAO needs to pay a greater cost *G* in order to crack down on fraudulent behavior.

(4) The MAO can punish the illegal acts of the MI and II, but the intensity of punishment φ is limited. To some extent, it can be determined by the strictness of its supervision.

(5) When the II actively carries out supervision and reports, the MAO can give it a certain reward (represented by *R*_3_) to encourage it to better play the role of social supervision. The II has no behavior cost, so its benefit is mainly related to the medical insurance reimbursement amount *R*_3_.

Based on the aforementioned assumptions, the relationship between the three participants can be obtained (see in Figure 1).

The notations used in the model and their interpretations are shown in Table 1.

**Table 1:** Notations and interpretations.

**Notations**	**Interpretations**
*C*_1_	Behavior cost of the medical administrative organization
*C*_2_	Behavioral cost of the medical institutions
*C*_3_	Normal reimbursement amount of the insured individuals
*q*_2_	Proceeds from fraud by the medical institutions
*q*_3_	Proceeds from medical fraud by the insured individuals
*R*_1_	Negative impact of fraudulent acquisitions on the medical administrative organization
*R*_2_	The penalties or rewards encountered by the medical institutions
*R*_3_	The penalties or rewards encountered by the insured individuals
*E*	Impact on the medical institutions when penalties are increased
*G*	The additional cost of strengthening supervision by the medical insurance administration
*m*	The benefits of collusive behavior to the medical institutions
*k*	The benefits of collusion to the insured individuals (*m*>*k*)
φ	The penalties of the medical administrative organization (0 < φ <1)
ω	Self-correction efforts of the medical institutions (0 < ω <1)
*x*	Probability of strategy of Medical Administration Organization
*y*	Probability of Strategy of Medical Institutions
*z*	Probability of Strategy of insured participants

*All the values are greater than zero*.

## Tripartite Evolutionary Game Model of Health Insurance Fraud

### Game Payment Matrix

Analyze the income of each subject in the game matrix under eight main cases.

#### The MAO Strengthens Supervision and Punishment

For the medical administrative organization, when strengthening supervision, its behavior cost is the sum of basic cost and additional cost (−*C*_1_−*G*). *C*_2_ is behavioral cost of medical institutions and *C*_3_ is the normal reimbursement amount of the insured individual. When the medical institutions and the insured individuals commit fraud, they are usually punished, so losses −φ*R*_2_ and −φ*R*_3_ may occur, respectively, and the fine becomes the income of the administrative department. The reward received by the insured individual for active reporting is *R*_3_. However, if the penalty is received, the fine is affected by the punishment intensity of the administrative department, so it is −ϕ*R*_3_. When medical institutions strictly abide by the regulations, due to the administrative department gives certain encouragement to the internal management construction of medical institutions, medical institutions generate benefits ω*R*_2_. When the medical institution colludes with the insured individuals, the designated medical institution obtains the income *m* and the individual obtains the income *k*. When the problem exposure is strengthened, and the designated medical institutions take fraud, it will cause reputation loss −*E*.

#### The MAO Relaxes Supervision

When the MAO relaxes the supervision, the probability of punishment for fraud and conspiracy between medical institutions and insured individuals is smaller, so they have greater motivation to collude and obtain collusion benefits *m* and *k*, respectively. It should be noted that, unlike the situation where the administrative department strengthens supervision, medical institutions and insured individuals are more likely to obtain benefits *q*_2_ and *q*_3_ through fraud. At the same time, it makes the MAO gain loss −*q*_2_−*q*_3_. In addition, the negative impact of fraud on the MAO (−*R*_1_) cannot be ignored. Therefore, the payment matrix of three game players can be obtained (shown in Table 2).

**Table 2:** Game payment matrix.

	**The MI abides by the regulations** (**y**)			**The MI defrauds health insurance** (**1−*y***)		
		**Revenue of MAO**	**Revenue of MI**	**Revenue of II**	**Revenue of MAO**	**Revenue of MI**	**Revenue of II**
The MAO strengthens supervision and punishment (*x*)	The II abide by the law (*z*)	−*C*_1_−*G*	−*C*_2_+ω*R*_2_	*C*_3_+*R*_3_	−*C*_1_−*G*	−*C*_2_−*E*−*R*_2_	*C*_3_+*R*_3_
	The II defrauded the health insurance (1−*z*)	−*C*_1_−*G*+φ*R*_3_	−*C*_2_+ω*R*_2_	−φ*R*_3_	−*C*_1_−*G*+φ*R*_2_+φ*R*_3_	−*C*_2_+*m*−*E*−φ*R*_2_	*k*−ϕ*R*_3_
The MAO relaxes supervision (1−*x*)	The II abide by the law (*z*)	0	−*C*_2_	*C*_3_+*R*_3_	−*C*_1_−*R*_1_	−*C*_2_+*m*+*q*_2_+*C*_3_	*R*_3_
	The II defrauded the health insurance (1−*z*)	−*R*_1_−*q*_3_	−*C*_2_	*C*_3_	−*C*_1_−*q*_2_−*q*_3_	−*C*_2_+*m*+*q*_2_	*C*_3_+*q*_3_+*k*

### Analysis of Equilibrium Strategy

Under different circumstances, the strategies of medical administrative organization, medical institutions and insured individuals will evolve in different directions. Therefore, it is necessary to first investigate the strategy evolution trend of each subject.

#### Evolutionary Equilibrium Strategy Trend of MAO

For the medical administrative organization, it is assumed that the expected benefits of the subject choosing “strengthened supervision” and “loose supervision” are *E*_*x*1_ and *E*_*x*2_, respectively, which can be calculated according to Table 2.


(1) $$\begin{array}{ll} E_{x1}=yz(-C_{1}-G)+(1-y)z(-C_{1}-G) & \;\\ +(1-z)y(-C_{1}-G+\varphi R_{3}\text{)} & \;\\ +(1-z)(1-y)(-C_{1}-G+\varphi R_{2}+\varphi R_{3}) & \;\\ \text{=}-C_{1}-G+(1-z)\left[\varphi R_{3}+(1-y)\varphi R_{2}\right] & \; \end{array}$$

(2) $$\begin{array}{l} E_{x2}=z(1-y)(-C_{1}-R_{1})+(1-z)y(-R_{1}-q_{3})\\ +(1-z)(1-y)(-C_{1}-q_{2}-q_{3}) \end{array}$$

Therefore, the replication dynamic equation of medical administrative organization is:


(3) $$\begin{array}{l} F_{1}(x)=\frac{dx}{dt}=x(1-x)(E_{x1}-E_{x2})\\ =x(1-x)\left\{\begin{array}{l} -C_{1}-G+(1-z)\left[\varphi R_{3}+(1-y)\varphi R_{2}\right]-z(1-y)(-C_{1}-R_{1})\\ -(1-z)y(-R_{1}-q_{3})-(1-z)(1-y)(-C_{1}-q_{2}-q_{3}) \end{array}\right\} \end{array}$$

According to the stability of the replication dynamic equation, and *F*_1_(*x*) = 0, we can obtain:


$$x=0,x=1,y^{x}=\frac{\begin{array}{l} C_{1}+G-(1-z)\varphi R_{3}-(1-z)\varphi R_{2}+z(-C_{1}-R_{1})\\ +y(-R_{1}-q_{3})+(1-z)(-C_{1}-q_{2}-q_{3}) \end{array}}{-\varphi R_{2}(1-z)-z(C_{1}+2R_{1}+q_{3})+(1-z)(-C_{1}-q_{2}-q_{3})}$$

**Figure 2:**
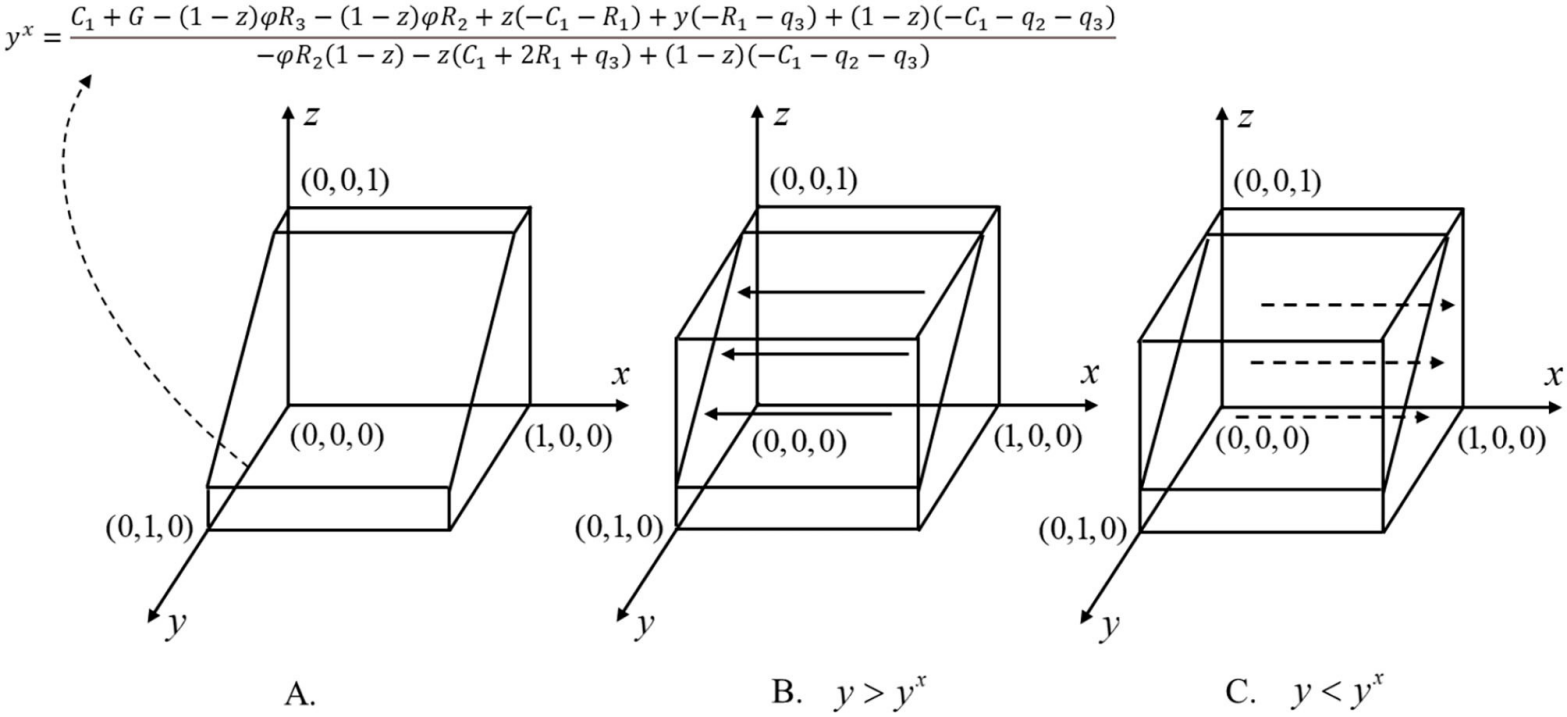
Dynamic evolution phase diagrams of MAO's strategy.

The aforementioned results are further described in the later sections. When *y* = *y*^*x*^, so *F*_1_(*x*) = 0, which means that for any *x*, this game is in a stable state, and the strategy of the MAO will not continue to evolve. When *y*≠*y*^*x*^, the condition of *F*_1_(*x*) = 0 is *x* = 0,*x* = 1. When *y*<*y*^*x*^, that is, the proportion of medical institutions choosing the strategy of “abiding by the regulations” is less than *y*^*x*^, we have *F*(*x*)>0, *F*′(1) <0, *F*′(0)>0, so *x* = 1 satisfies the stable equilibrium condition. *x* = 1 is the stable equilibrium of the medical administrative organization, which shows that when medical institutions are more inclined to “defraud health insurances”, the strategy of the administrative department will eventually evolve to “strengthen supervision”; on the contrary, when *y*>*y*^*x*^, that is, the proportion of the medical institutions choosing the strategy of “compliance with laws and regulations” is greater than *y*^*x*^, we can obtain *F*(*x*) <0, *F*′(1)>0, *F*′(0) <0. At this time, the stable equilibrium is *x*=0, which means when the strategy of the medical institutions tends to “abide by the law”, the strategy of the government will eventually evolve to “deregulation”. Figure 2 shows the dynamic evolution phase diagram of the decision-making of the MAO.

#### Evolutionary Equilibrium Strategy Trend of MI

Similar to the aforementioned analysis process, for the MI, it is assumed that the expected returns of this subject choosing “compliance with laws and regulations” and “defrauding Health insurances” are *E*_*y*1_ and *E*_*y*2_, respectively, which can be calculated according to Table 2.


(4) $$\begin{array}{ll} E_{y1}=xz(-C_{2}+\omega R_{2})+x(1-z)(-C_{2}+\omega R_{2}) & \;\\ +(1-x)z(-C_{2}\text{)}+(1-x)(1-z)(-C_{2}) & \;\\ =x(-C_{2}+\omega R_{2})+(1-x)(-C_{2}\text{)} & \; \end{array}$$

(5) $$\begin{array}{l} E_{y2}\text{​ }=\text{​ }xz(-C_{2}-E-R_{2})\text{​ }+\text{ ​}x(1-z)(-C_{2}\text{​ }+\text{ ​}m-E-\varphi R_{2})\text{​}\\ +\text{​}(1-x)z(-C_{2}\text{​ }+\text{ ​}m\text{ ​}+\text{ ​}q_{2}\text{​}+\text{ ​}C_{3}\text{) ​}+\text{ ​}(1-x)(1-z)(-C_{2}\text{​ }+\text{ ​}m\text{ ​}+\text{ ​}q_{2})\\ =\text{​}x(-C_{2}-E)-xz(R_{2}\text{​ }+\text{ ​}C_{3})\text{ ​}+\text{ ​}x(1-z)(m-\varphi R_{2})\text{​ }+\text{ ​}(1-x)\\ (-C_{2}\text{​ }+\text{ ​}m\text{ ​}+\text{ ​}q_{2})\text{ ​}+\text{ ​}zC_{3} \end{array}$$

Hence, the replication dynamic equation of MI is shown as Equation (6).


(6) $$\begin{array}{ll} F_{2}(y)=\frac{dy}{dt}=y(1-y)(E_{y1}-E_{y2}) & \;\\ \text{​}=\text{​}y(1-y)\left[\begin{array}{l} x\omega R_{2}+(1-x)(-C_{2})-xE+xz(R_{2}+C_{3})\\ -x(1-z)(m-\varphi R_{2})-(1-x)(-C_{2}+m+q_{2})-zC_{3} \end{array}\right]\text{​​} & \; \end{array}$$

According to the stability of the replication dynamic equation, and *F*_2_(*y*) = 0, we can obtain:


$$y=0,y=1,x^{y}=\frac{zC_{3}+m+q_{2}}{\omega R_{2}-E+z(R_{2}+C_{3})-(1-z)(m-\varphi R_{2})+m+q_{2}}$$

Similarly, if *x*<*x*^*y*^, which means that the proportion of the strategy of “strict supervision” selected by the MAO is less than *x*^*y*^, *F*(*y*)>0, *F*′(1)>0, *F*′(0) <0 can be obtained, and the equilibrium result is *y*=0. It shows that when the MAO is more inclined to “relax supervision”, the strategy of medical institutions will eventually evolve to “defraud health insurance”; on the contrary, when *x*>*x*^*y*^, the proportion of “strictly abide by the regulations” selected by the MAO will greater than *x*^*y*^, and there are *F*(*y*)>0, *F*′(1) <0, *F*′(0)>0. At this time, the stable equilibrium of MAO is *y*=1. This result shows that when the strategy of the MAO tends to “Strict Supervision”, the strategy of MI will eventually evolve to “Do not take fraud”.

#### Evolutionary Equilibrium Strategy Trend of II

For the insured individuals, it is assumed that the expected income of “strictly abiding by relevant laws and regulations” and “defrauding Health insurance” are *E*_*z*1_ and *E*_*z*2_, respectively, Equations (7) and (8) can also be calculated according to Table 2. And the replication dynamic equation of insured individual is shown as Equation (9).


(7) $$\begin{array}{l} E_{z1}=xy(C_{3}+R_{3})+x(1-y)(C_{3}+R_{3})\\ +(1-x)y(C_{3}+R_{3}\text{)}+(1-x)(1-y)R_{3}\\ =R_{3}+(x+y-xy)C_{3} \end{array}$$

(8) $$\begin{array}{ll} E_{z2}=xy(-\varphi R_{3})+x(1-y)(k-\varphi R_{3})+(1-x)yC_{3} & \;\\ +(1-x)(1-y)(C_{3}+q_{3}+k) & \;\\ =x(k-\varphi R_{3})-xyk+(1-x)C_{3}+(1-x)(1-y)(q_{3}+k) & \; \end{array}$$

(9) $$\begin{array}{ll} F_{3}(z)=\frac{dz}{dt}=z(1-z)(E_{z1}-E_{z2}) & \;\\ =z(1-z)R_{3}+(y-xy)C_{3}-x(k-\varphi R_{3})+xyk+C_{3} & \;\\ -(1-x)(1-y)(q_{3}+k)] & \; \end{array}$$

According to the stability of the replication dynamic equation, and *F*_3_(*z*) = 0, we can obtain:


$$z=0,z=1,y^{z}=\frac{-R_{3}+x(k-\varphi R_{3})-C_{3}+(1-x)(q_{3}+k)}{(1-x)C_{3}+q_{3}+k-xq_{3}}$$

If *y*<*y*^*z*^ and when the proportion of MI choosing the strategy of “abide by the regulations” is less than*y*^*z*^, we have *F*(*z*)>0, *F*′(1)>0, *F*′(0) <0, so the stable equilibrium is *z*=0. It shows that when the medical institutions are more inclined to “defraud health insurance”, the strategy of the insured individuals will eventually evolve to “defraud health insurance”; on the contrary, if *y*>*y*^*z*^, which means the proportion of MI choosing the strategy of “strictly abide by the regulations” is more than*y*^*z*^, we have *F*(*y*)>0, *F*′(1) <0, *F*′(0)>0, so the stable equilibrium of the insured individual is *z*=1. The aforementioned analysis shows that when the strategy of the medical institutions tends to “strictly abide by the regulations”, the strategy of insured individuals will also evolve to “abide by the regulations”.

It should be pointed out that the aforementioned analysis only considers the impact of the initial strategy probability on the strategy evolution trend of each subject, which belongs to the analysis of local evolution trend. Moreover, because it focuses on the influence of one party on the other, the evolutionary stability strategy (ESS) of the whole system has not been revealed. It is necessary to further analyze the possible equilibrium points and their asymptotic stability.

### Stability Point and Its Asymptotic Stability

After investigating the strategy evolution trend of each subject under specific conditions, it is necessary to further analyze the asymptotic stability of the equilibrium point of the system, so as to obtain the evolutionary stability strategy (ESS) of the whole system. In the replication dynamic system of the three subjects, the stability of participant strategies can be judged by *Lyapunov's first law*.*x*(*t*), *y*(*t*), *z*(*t*)∈[0, 1] is used to represent the dynamic probability of each game subject over time. For each initial point (*x*(*t*), *y*(*t*), *z*(*t*))∈[0, 1] × [0, 1] × [0, 1], any point (*x, y, z*) in the solution set of tripartite evolutionary game strategy corresponds to the mixed strategy equilibrium of an evolutionary strategy. The possible evolutionary equilibrium point can be solved by the three main replication dynamic equations, see Equation (10).


(10) $$\{\text{​​}\begin{array}{ll} F_{1}(x)=\frac{dx}{dt}=x(1-x)(E_{x1}-E_{x2})= & \;\\ x(1-x)\left\{\begin{array}{l} -C_{1}-G+(1-z)\left[\varphi R_{3}+(1-y)\varphi R_{2}\right]-z(1-y)(-C_{1}-R_{1})\\ -(1-z)y(-R_{1}-q_{3})-(1-z)(1-y)(-C_{1}-q_{2}-q_{3}) \end{array}\text{​​}\right\}\text{=}0\text{​​} & \;\\ F_{2}(y)=\frac{dy}{dt}=y(1-y)(E_{y1}-E_{y2})= & \;\\ y(1-y)\left[\begin{array}{l} x\omega R_{2}+(1-x)(-C_{2})-xE+xz(R_{2}+C_{3})-\\ x(1-z)(m-\varphi R_{2})-(1-x)(-C_{2}+m+q_{2})-zC_{3} \end{array}\right]\text{ =}0 & \;\\ F_{3}(z)=\frac{dz}{dt}=z(1-z)(E_{z1}-E_{z2})= & \;\\ z(1-z)\left[\begin{array}{l} R_{3}+(y-xy)C_{3}-x(k-\varphi R_{3})+xyk\\ +C_{3}-(1-x)(1-y)(q_{3}+k) \end{array}\right]\text{ =}0 & \; \end{array}$$

By solving Equation (10), we can get eight equilibrium points: *E*1 (0,0,0), *E*2 (1,0,0), *E*3 (0,1,0), *E*4 (0,0,1), *E*5 (1,1,0), *E*6 (1,0,1), *E*7 (0,1,1) and *E*8 (1,1,1). These equilibrium points are actually located at the boundary of the set of tripartite evolutionary game strategies, that is {(*x, y, z*)|*x* = 0, 1;*y* = 0, 1;*z* = 0, 1}, and the interior of a policy set can be expressed as:


Ω={(x,y,z)|0<x<1,0<y<1,0<z<1}

Furthermore, for the ninth equilibrium point *E*9(*y*^*x*^, *x*^*y*^, *y*^*z*^) that should be located in the solution set, if *E*9∉Ω, the point should be discarded.

In a multi-agent evolutionary game, the asymptotic stable equilibrium point must be an ESS. Hence, in a tripartite evolutionary game, if an equilibrium point is an asymptotic stable point, it must be a pure strategic Nash equilibrium. Since *E*9(*y*^*x*^, *x*^*y*^, *x*^*z*^) is a mixed strategy Nash equilibrium, it can be judged that *E9* is not an asymptotic stability point, and only the asymptotic stability of the other eight points needs to be investigated. The Jacobian matrix of the aforementioned tripartite evolutionary game is shown as Equation (11).


(11) $$J\text{=}\left\{\begin{array}{ccc} \begin{array}{ll} \frac{\partial F_{1}(x)}{\partial x} & \;\\ \; & \frac{\partial F_{2}(y)}{\partial x}\\ \; & \frac{\partial F_{3}(z)}{\partial x} \end{array} & \begin{array}{ll} \frac{\partial F_{1}(x)}{\partial y} & \;\\ \; & \frac{\partial F_{2}(y)}{\partial y}\\ \; & \frac{\partial F_{3}(z)}{\partial y} \end{array} & \begin{array}{ll} \frac{\partial F_{1}(x)}{\partial z} & \;\\ \; & \frac{\partial F_{2}(y)}{\partial z}\\ \; & \frac{\partial F_{3}(z)}{\partial z} \end{array} \end{array}\right\}$$

Among this:


$$\begin{array}{l} \frac{\partial F_{1}(x)}{\partial x}\text{=}(1-2x)\\ \left\{\begin{array}{l} -C_{1}-G+(1-z)\left[\varphi R_{3}+(1-y)\varphi R_{2}\right]-z(1-y)(-C_{1}-R_{1})-\\ \left(1-z)y(-R_{1}-q_{3})-(1-z)(1-y)(-C_{1}-q_{2}-q_{3}\right) \end{array}\right\} \end{array}$$

$$\frac{\partial F_{1}(x)}{\partial y}\text{​ }=\text{ ​}x(1-x)\text{​}\left[\text{​}-z(C_{1}+R_{1})+(1-z)(R_{1}-\varphi R_{2}-C_{1}-q_{2})\text{​}\right]\text{​}$$

$$\begin{array}{l} \frac{\partial F_{1}(x)}{\partial z}=x(1-x)\\ \left[-\varphi R_{3}-(1-y)\varphi R_{2}+y(-R_{1}-q_{3})+(1-y)(R_{1}-q_{2}-q_{3})\right] \end{array}$$

$$\begin{array}{l} \frac{\partial F_{2}(y)}{\partial x}=y(1-y)\\ \left[\omega R_{2}-E+z(R_{2}+C_{3})-(1-z)(m-\varphi R_{2})+m+q_{2}\right] \end{array}$$

$$\begin{array}{l} \frac{\partial F_{2}(y)}{\partial y}\text{=}(1-2y)\\ \left[\begin{array}{l} x\omega R_{2}+(1-x)(-C_{2})-xE+xz(R_{2}+C_{3})\\ -x(1-z)(m-\varphi R_{2})-(1-x)(-C_{2}+m+q_{2})-zC_{3} \end{array}\right] \end{array}$$

$$\frac{\partial F_{2}(y)}{\partial z}=y(1-y)\left[x(R_{2}+C_{3})+x(m-\varphi R_{2})-C_{3}\right]$$

$$\frac{\partial F_{3}(z)}{\partial x}=z(1-z)\left[-y(q_{3}+C_{3})+\varphi R_{3}+q_{3}\right]$$

$$\frac{\partial F_{3}(z)}{\partial y}=z(1-z)\left[(1-x)C_{3}+q_{3}+k-xq_{3}\right]$$

$$\begin{array}{l} \frac{\partial F_{3}(z)}{\partial z}=(1-2z)[R_{3}+(y-xy)C_{3}-x(k-\varphi R_{3})+xyk+C_{3}\\ \;-(1-x)(1-y)(q_{3}+k)] \end{array}$$

Based on Jacobian matrix, the asymptotic stability of eight stable points can be analyzed. Only when the terms of the main diagonal of Jacobian matrix are negative, the point is an equilibrium point. Table 3 lists the asymptotic stability analysis of the eight points. If a point is unstable, the reasons are given; if the conditions at a certain point are stable, the conditions that should be met are given.

**Table 3:** Equilibrium points and their asymptotic stability.

**Point**	**Stability**	**Reasons or conditions**
(0,0,0)	Conditional stable	φ(*R*_2_+*R*_3_)+*q*_2_+*q*_3_<*G, R*_3_+*C*_3_<*q*_3_+*k*
(1,0,0)	Conditional stable	φ(*R*_2_+*R*_3_)+*q*_2_+*q*_3_>*G*, ω*R*_2_+φ*R*_2_<*E*+*m*
(0,1,0)	Unstable	$\frac{\partial F\left(y\right)}{\partial y}>0$
(0,0,1)	Conditional stable	*R*_1_<*G, R*_3_+*C*_3_>*q*_3_+*k*
(1,1,0)	Unstable	$\frac{\partial F\left(z\right)}{\partial z}>0$
(1.0,1)	Conditional stable	*G*<*R*_1_, ω*R*_2_+*R*_2_<*E, R*_3_+φ*R*_3_+*C*_3_>*k*
(0,1,1)	Unstable	$\frac{\partial F\left(y\right)}{\partial y}>0$
(1,1,1)	Unstable	$\frac{\partial F\left(x\right)}{\partial x}>0$

Through the asymptotic stability analysis of the possible equilibrium point, it can be found that there are four conditional stable points and four unstable points. For equilibrium points (0,0,0), (1,0,0), (0,0,1), (1,0,1), it can achieve asymptotic stability when certain conditions are met. Next, the actual and economic meaning of the aforementioned four equilibrium points is analyzed.

#### Point (0,0,0)

The actual meaning of this point is that the medical administrative organization implements loose supervision, and the medical institutions and the insured individuals have a strong motivation to defraud the health insurance. In this case, the fraud of health insurance will not be curbed, but will become more serious, and eventually seriously damage the interests of all subjects. Therefore, it is a serious result that should be vigilant. In the absence of strong supervision by the medical administrative organization, the other two parties may not only seek benefits for themselves alone, but also cheat more health insurance through collusion. Therefore, the medical institutions no longer have the motivation to self-supervise and self-correct, and the insured individuals will not supervise and report.

#### Point (1,0,0)

For point (1,0,0), its actual meaning is that although the medical administrative organization carries out strict supervision and takes the behavior of increasing exposure and punishment, the medical institutions and insured individuals still intend to defraud the health insurance. In fact, the goal of the medical administrative department needs not only to increase punishment, but also to guide the medical institutions to improve the internal supervision mechanism and guide the insured individuals to actively play the role of supervision and reporting through a certain degree of reward. This means that in the evolutionary game, the values of *R*_2_, *R*_3_ should be large.

#### Point (0,1,1)

For point (0,0,1), it means that only the insured individuals will strictly abide by the regulations, while the medical institutions attempt to defraud. At the same time, loose supervision of the MAO will also lead to this phenomenon to a certain extent. Medical insurance participants are often in a weak position in the whole game system. They not only face the limited amount of medical insurance reimbursement, but also need to pay more financial resources once their interests are infringed. This phenomenon enlightens that governments need to focus on protecting the interests of insured individuals.

#### Point (1,0,1)

For point (1,0,1), it means that the medical administrative organization implements strict supervision and the insured person can comply with relevant regulations, but the medical institutions may still choose the strategy of “defraud the health insurance fund”. In practice, the problem of defrauding health insurance mainly occurs in medical institutions. Therefore, how to stop the fraud from the medical institutions is the core of the whole problem. To standardize the behavior of medical institutions, we need the combination of external supervision, supervision and internal self-inspection, and self-correction.

## Simulation Analysis

In order to reveal the game results produced by the supervision strategy of MAO, the strategy evolution trend of each game subject can be intuitively displayed through simulation analysis. In each time interval, the three subjects participating in the game formulate a strategy to meet their expected returns. Then, each participant revises their strategy according to the results of the previous time interval (Δ*t*-1). By using the discrete equations and repeating the iterative calculation for many times, the asymptotic evolution stability of all parties can be obtained. This section mainly simulates and analyzes the aforementioned four situations and the influence of main parameters.

### ESS (0,0,0)

In fact, many health insurance frauds occur in China every year. Based on the case analysis and data statistics, the values of parameters which used in the simulation analysis can be obtained. For example, to analyze the ESS(0,0,0), we find a case that the medical administrative organization relaxes supervision and the medical institutions and the insured individuals take the fraud strategy. In this case, we accurately measured the benefits and losses of the participants.

**Figure 3:**
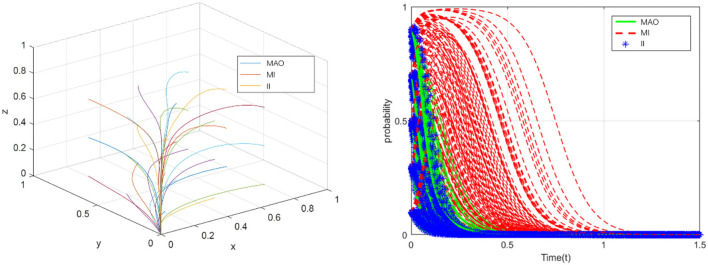
The evolution toward the sink ESS (0,0,0).

Hence, based on the analysis of the operation of China's health insurance and in combination with the conditions that should be met to achieve equilibrium, that is, φ(*R*_2_+*R*_3_)+*q*_2_+*q*_3_<*G, R*_3_+*C*_3_<*q*_3_+*k*, we suppose that *C*_1_ = 16, *C*_2_ = 15, *C*_3_ = 13, *R*_1_ = 12, *R*_2_ = 9, *R*_3_ = 5, *G* = 23, *q*_1_ = 11, *q*_2_ = 6, *q*_3_ = 17, ω = 0.5, φ = 0.5, *E* = 7, *m* = 5, *k* = 2. For each initial probability value (*x*(0), *y*(0), *z*(0)), the medical administrative organization tends to relax supervision, which will lead to the fraud of the medical institutions and the insured individuals (see in Figure 3). The simulation results show that the evolutionary stability strategy will be (0,0,0) when the sum of rewards or fraud benefits obtained by the medical institutions and the insured individuals (φ*R*_2_+φ*R*_3_) is less than the additional benefits of strengthening government supervision (*q*_2_+*q*_3_), and the legitimate benefits of the insured individuals (*R*_3_+*C*_3_) are less than the sum of fraud benefits and collusion benefits (*q*_3_+*k*). In this case, the problem of fraud of health insurance has not been solved, and eventually damage the interests of all parties.

### ESS (1,0,0)

**Figure 4:**
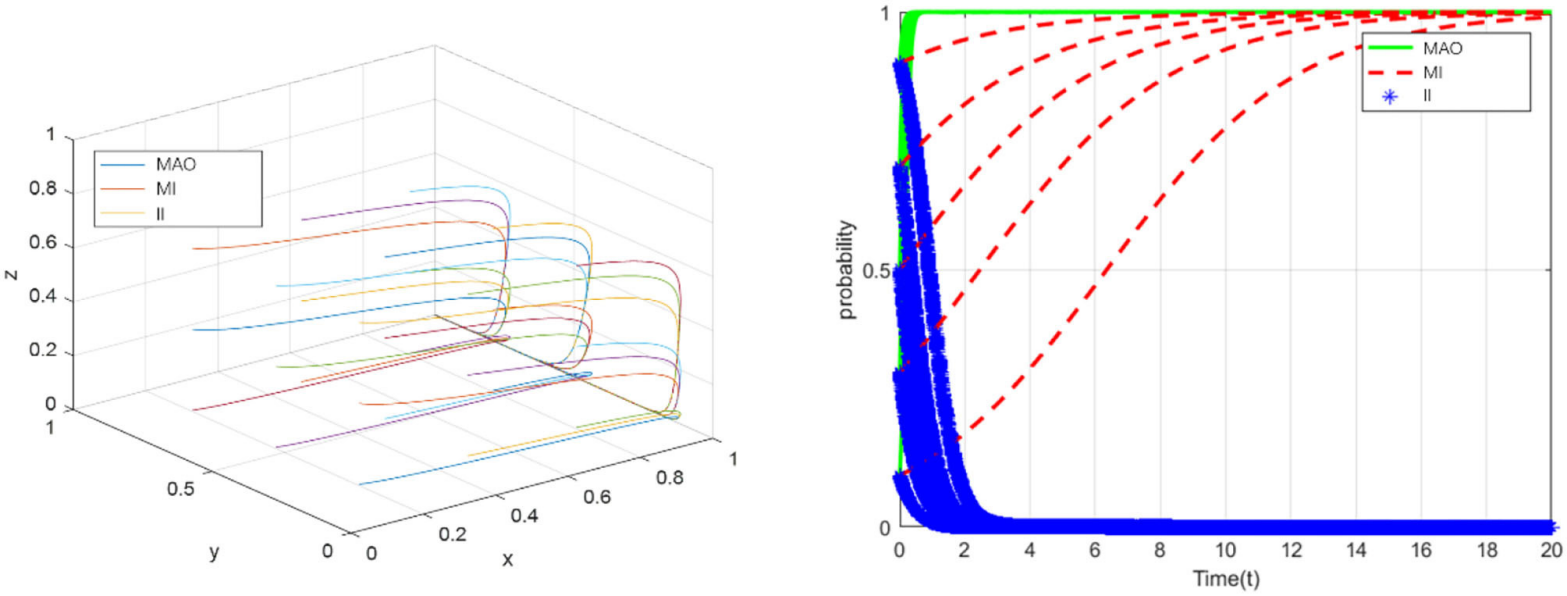
The evolution toward the sink ESS (1,0,0).

Analyze the conditions that should be met when the point reaches equilibrium,and suppose that*C*_1_ = 18, *C*_2_ = 10, *C*_3_ = 11, *R*_1_ = 10, *R*_2_ = 9, *R*_3_ = 5, *G* = 18, *q*_1_ = 5, *q*_2_ = 6, *q*_3_ = 15, ω = 0.5, φ = 0.5, *E* = 8, *m* = 4, *k* = 2. For each initial probability value (*x*(0), *y*(0), *z*(0)), although the medical administrative organization takes strict regulatory measures, the medical institutions and the insured individuals will still take fraud (see in Figure 4). The simulation results show that, under the condition of φ(*R*_2_+*R*_3_)+*q*_2_+*q*_3_>*G*, ω*R*_2_+φ*R*_2_<*E*+*m*, the evolutionary stable point will be (1,0,0). This means that even if the medical administrative organization adopts the strategy of strengthening supervision, under certain conditions, medical institutions and individuals will still choose the strategy of “defraud the health insurance fund”, so the health insurance cannot achieve healthy and sustainable development. ESS (0,0,0) and ESS (1,0,0) jointly explain that strengthening supervision and increasing punishment by the medical administrative organization could still not solve the problem. The policies of the medical regulatory department need to take into account the standards of punishment and reward, and carefully evaluate the benefits generated by the collusion of the medical institutions and the insured individuals.

### ESS (0,0,1)

**Figure 5:**
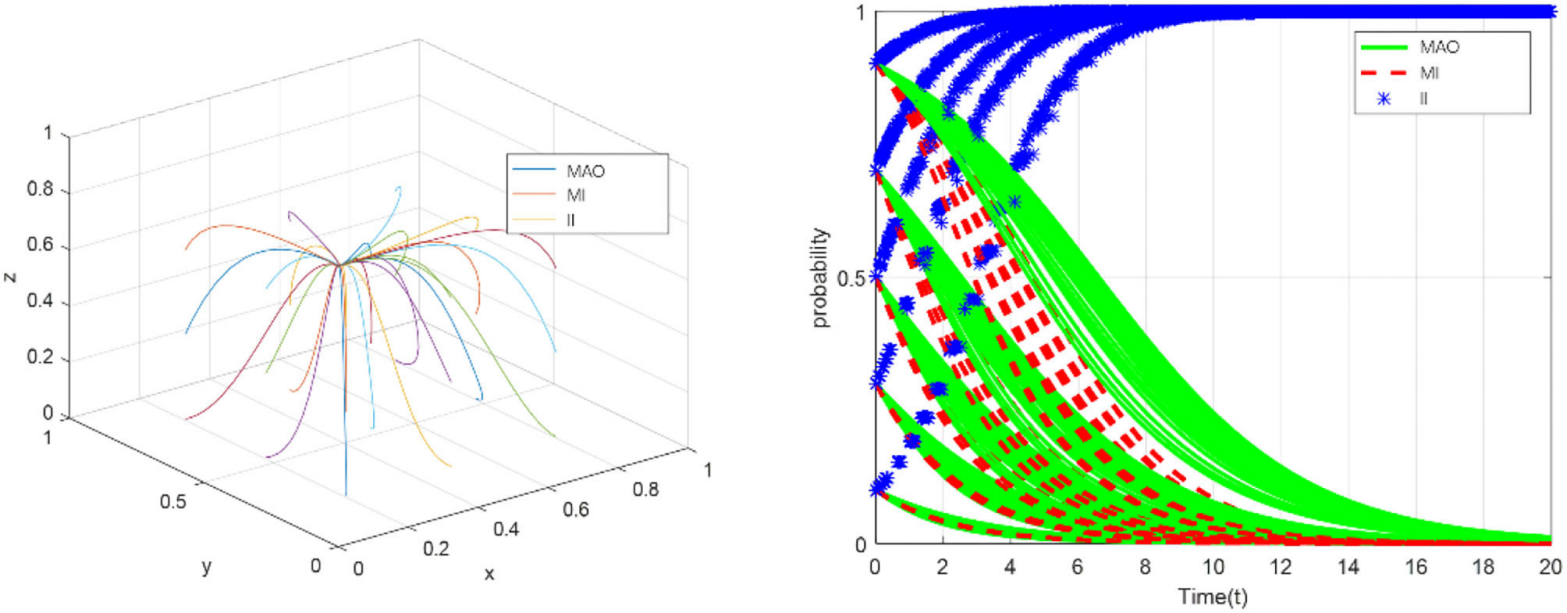
The evolution toward the sink ESS (0,0,1).

According to the conditions that should be met to reach equilibrium at point (0,0,1), suppose that *C*_1_ = 21, *C*_2_ = 14, *C*_3_ = 11, *R*_1_ = 9, *R*_2_ = 7, *R*_3_ = 3.5, *G* = 26, *q*_1_ = 33, *q*_2_ = 16, *q*_3_ = 10, ω = 0.3, φ = 0.3, *E* = 7, *m* = 3, *k* = 2. In this case, the medical administrative organization does not take strict regulatory measures, and the medical institutions strictly complied with laws and regulations, only the insured individuals intended to take fraud (see in Figure 5). The simulation results show that, under the condition of *R*_1_<*G, R*_3_+*C*_3_>*q*_3_+*k*, the ESS will be (0,0,1). Because the insured person is usually in the relatively weak side in the tripartite game, they often choose to abide by the regulations to obtain reimbursement. If the insured individual commits fraud, they may not only lose the original reimbursement amount, but also face serious consequences such as multiple penalties. The lack of professionalism and fraud ability of the insured individual is also one of the reasons. The enlightenment of this to the medical administrative organization mainly has the following two points. First, the insured individuals are more motivated to comply with the regulations. The medical administrative organization can further give full play to the extensive supervision role of the insured individuals and jointly build a supervision network for medical institutions. Second, the insured person is more likely to become the victim of fraud. Hence, the medical regulatory authorities should pay attention to the protection of the interests of this group and identifying the infringement of the interests of this group through the medical institutions.

### ESS (1,0,1)

**Figure 6:**
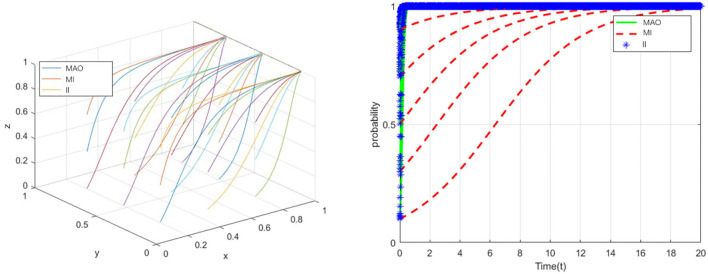
The evolution toward the sink ESS (1,0,1).

Similarly, according to the conditions that should be met to reach equilibrium at point (1,0,1), suppose that*C*_1_ = 25, *C*_2_ = 11, *C*_3_ = 12, *R*_1_ = 10, *R*_2_ = 12, *R*_3_ = 5, *G* = 8, *q*_1_ = 11, *q*_2_ = 4.5, *q*_3_ = 13, ω = 0.6, φ = 0.6, *E* = 21, *m* = 7, *k* = 13. In this case, the medical administrative organization will take strict regulatory measures, the insured individuals will strictly abide by relevant laws and regulations, and only medical institutions intend to defraud health insurance fund (see in Figure 6). The simulation results illustrate that, under the condition of *G*<*R*_1_, ω*R*_2_+*R*_2_<*E, R*_3_+φ*R*_3_+*C*_3_>*k*, the ESS will be point (1,0,1). From a practical point of view, ESS (1,0,1) is a relatively ideal result compared with the aforementioned three results. Although the medical institutions will still take fraud, that is, the problem has not been fundamentally solved, it further proves that medical institutions are the difficulty and core of the whole problem. The strict supervision of the medical administrative organization will further promote the insured individuals to choose the strategy of “comply with the regulations” and actively report, but the medical institutions still have a strong motivation to cheat the health insurance, and the collusion with the insured individuals will not become a necessary condition for the medical institutions to break the law.

## Conclusions

In the process of operation and supervision of China's health insurance, there are three types of subjects: medical administrative organizations, medical institutions, and insured individuals. By analyzing the interest structure of multi-agent and the game relationship between them, this article constructs an evolutionary game model, and analyses the four evolutionary stability points of the system through the dynamic equations. The simulation analysis further investigates the practical reasons for the formation of evolutionary stability points. Based on the aforementioned analysis, the main conclusions of this study are as follows.

In the absence of strong supervision by the medical administrative organization, the medical institutions and the insured individuals may not only seek benefits for themselves alone, but also cheat more health insurance through collusion. Therefore, the medical institutions no longer have the power to conduct self-examination and self-correction, and insured individuals will not supervise and report. At the same time, the medical administrative organization should make it clear in the relevant policies that the problem cannot be solved by strengthening supervision and punishment. The policies of the regulatory authorities need to take into account the standards of punishment and reward, and carefully evaluate the benefits generated by the collusion of the medical institutions and the insured individuals.

In the tripartite game of health insurance, the insured individuals are usually in a relatively weak party. Therefore, they tend to abide by the regulations in order to obtain medical insurance reimbursement. Based on this, this article believes that the medical administrative department can further give full play to the extensive supervision role of the insured individuals and jointly build a supervision network to medical institutions. At the same time, the medical regulatory authorities should pay attention to the protection of the interests of vulnerable groups and identify the infringement of the interests of this group by medical institutions.

Strict supervision of the medical administrative organization will further promote the insured individuals to choose “abide by the regulations” and actively report. However, the medical institutions still have a strong motivation to defraud health insurance, and collusion with the insured individuals will not become a necessary condition for the medical institutions. Therefore, strengthening the supervision of the medical institutions is the core to solve the problem of fraud and compensation of health insurance fund.

## Suggestions for Government

Based on the research conclusion, the following measures and suggestions are put forward to solve the problem of health insurance fraud.

First, organize professional forces to strengthen the supervision of the medical institutions. The equilibrium analysis of the evolutionary model shows that when the medical administrative institutions strengthen supervision and the insured individuals actively report, the medical institutions may still take deception, because the medical institutions have more professional means of fraud and deception, so they are easier to obtain illegitimate interests by fabricating false treatment projects and excessive treatment. Therefore, it is necessary to organize professional forces, strengthen the recruitment, and improve the professional ability of inspectors. Build the internal management system of health insurance in medical institutions to effectively use health insurance. And further strengthen the operation and management of medical institutions, standardize medical service behavior, effectively control medical expenses, and maximize the occurrence of exceeding the standard and refusing to pay of health insurances (21).

Second, improve the regulatory mechanism for the multiple subjects and expand the regulatory coverage. Build a supervision mechanism with the participation of multiple subjects and a supervision mechanism combining internal management and external supervision. Meanwhile, the government can improve the ability to identify fraud by introducing a third-party regulatory body. Strengthen social supervision, formulate and improve the social supervision administrator system, clarify the reward for reporting violations, and enhance the effectiveness of social supervision. Meanwhile, fully realize information sharing and implement the whole process management of the fund use by using big data, Internet and other technologies (22). The regulatory authorities should actively publicize the policies to the medical institutions and the insured individuals, so as to reduce the probability of health insurance fraud.

Last but not least, it is of great significance to optimize the punishment mechanism for deception and establish an incentive mechanism for self-inspection and self-correction of medical institutions. Furthermore, improve the legal system of health insurance fund supervision, and clarify the punishment methods and standards. The medical administrative department should punish illegal acts according to related regulations, and establish a multi-dimensional punishment mechanism for health insurance fraud. Establish an integrity evaluation system, dynamically evaluate the medical insurance reimbursement behavior of the medical institutions, regularly assess the service content of medical institutions, and create a good atmosphere for legal operation. At the same time, try to include cheating in individual's integrity files to improve the authority of integrity result evaluation.

## Data Availability Statement

The original contributions presented in the study are included in the article/supplementary material, further inquiries can be directed to the corresponding author/s.

## Author Contributions

YFe: formal analysis and writing—review and editing. YFu: funding acquisition, D-xY: formal analysis, investigation, and methodology. C-hH: formal analysis, funding acquisition, writing—review and editing, software, and investigation. All authors contributed to the article and approved the submitted version.

## Conflict of Interest

The authors declare that the research was conducted in the absence of any commercial or financial relationships that could be construed as a potential conflict of interest.

## Publisher's Note

All claims expressed in this article are solely those of the authors and do not necessarily represent those of their affiliated organizations, or those of the publisher, the editors and the reviewers. Any product that may be evaluated in this article, or claim that may be made by its manufacturer, is not guaranteed or endorsed by the publisher.
